# Effects of Prestretch on Neonatal Peripheral Nerve: An *In Vitro* Study

**DOI:** 10.1055/s-0042-1743132

**Published:** 2022-04-08

**Authors:** Anita Singh, Tanmay Majmudar, Rachel Magee, Bernard Gonik, Sriram Balasubramanian

**Affiliations:** 1 Department of Biomedical Engineering, Widener University School of Engineering, Chester, Pennsylvania, United States; 2 Drexel University School of Biomedical Engineering, Science, and Health Systems, Philadelphia, Pennsylvania, United States; 3 Drexel University College of Medicine, Philadelphia, Pennsylvania, United States; 4 Department of Obstetrics and Gynecology, Wayne State University School of Medicine, Detroit, Michigan, United States

**Keywords:** neonates, brachial plexus, prestretch, tibial nerve, stretch, injury

## Abstract

**Background** Characterizing the biomechanical failure responses of neonatal peripheral nerves is critical in understanding stretch-related peripheral nerve injury mechanisms in neonates.

**Objective** This in vitro study investigated the effects of prestretch magnitude and duration on the biomechanical failure behavior of neonatal piglet brachial plexus (BP) and tibial nerves.

**Methods** BP and tibial nerves from 32 neonatal piglets were harvested and prestretched to 0, 10, or 20% strain for 90 or 300 seconds. These prestretched samples were then subjected to tensile loading until failure. Failure stress and strain were calculated from the obtained load-displacement data.

**Results** Prestretch magnitude significantly affected failure stress but not the failure strain. BP nerves prestretched to 10 or 20% strain, exhibiting significantly lower failure stress than those prestretched to 0% strain for both prestretch durations (90 and 300 seconds). Likewise, tibial nerves prestretched to 10 or 20% strain for 300 seconds, exhibiting significantly lower failure stress than the 0% prestretch group. An effect of prestretch duration on failure stress was also observed in the BP nerves when subjected to 20% prestretch strain such that the failure stress was significantly lower for 300 seconds group than 90 seconds group. No significant differences in the failure strains were observed. When comparing BP and tibial nerve failure responses, significantly higher failure stress was reported in tibial nerve prestretched to 20% strain for 300 seconds than BP nerve.

**Conclusion** These data suggest that neonatal peripheral nerves exhibit lower injury thresholds with increasing prestretch magnitude and duration while exhibiting regional differences.

## Introduction

Peripheral nerves can withstand physiological stretch under normal conditions, but abnormal stretch durations at strains within and beyond physiological limits can induce changes within the tissue that could predispose it to injury. 1 2 Available studies on peripheral nerves primarily focus on resulting functional and structural changes when subjected to varying degrees of stretch. 1 3 4 5 6 7 8 Most nerve prestretch (within physiological limits) studies investigate the effects of rate of stretching during repair of transected nerves. 9 No study has investigated the effects of prestretch magnitude while including non-physiological stretch limits and duration on the biomechanical behavior of nerve tissue thereby limiting our understanding of nerve injury mechanisms in prestretched peripheral nerves.

Peripheral nerve injuries can occur in individuals of all ages, including neonates. Existing knowledge of mechanical thresholds of peripheral nerve injury during stretch is obtained from studies in adult animal and human cadaveric tissue. 10 11 12 13 14 15 16 17 A recent study by Singh et al 18 utilized a neonatal piglet animal model to report the mechanical failure responses of brachial plexus (BP) and tibial nerves. This study also reported the rate-dependent behavior of the neonatal peripheral nerves. While this and other available studies in adults offer an understanding of the biomechanical properties of peripheral nerves, 19 20 there are no studies that have investigated changes in the biomechanical failure behavior of nerve tissue when subjected to physiological, as well as non-physiological, prestretch for varying durations that occurs during events leading to peripheral nerve injuries.

Shoulder dystocia is a complicated birthing scenario that is often accompanied by prolonged delivery times and may result in birthing brachial plexus injury (BBPI) that has a reported occurrence of 1.2 to 2.2 per 1,000 live births. 21 While the reported injury is a result of non-physiological strains on the BP, there is currently no study that has investigated the effects of prestretch magnitude and duration that account for the amount of neck stretch and delivery time during shoulder dystocia, respectively, on the biomechanical behavior of neonatal BP tissue. The magnitude and duration of prestretch, alone or together, might play a critical role in the observed injury. Therefore, characterizing mechanical injury thresholds in prestretched neonatal peripheral nerves is critical to better understand the nerve injury mechanisms.

Neonatal nerves, like adult peripheral nerves, must undergo stress relaxation, a property of viscoelastic materials that results in a time-dependent reduction in stress after the material has been stretched and held for a period of time. Accordingly, this study aims to provide the failure responses of peripheral nerves (BP and tibial), using a neonatal porcine animal model immediately after stress relaxation at two different prestretch strain levels (10 and 20%) and durations (90 and 300 seconds). Characterizing nerve failure responses under various prestretch durations and strain levels can offer a better understanding of nerve injury mechanisms that may help develop and improve injury prevention strategies.

## Materials and Methods

### Tissue Harvest

A total of 102 BP and 48 tibial nerve samples were obtained immediately postmortem from 32 neonatal piglets (3–5 days old). Using an axillary approach, the lower three cervical (C6–8) and first thoracic (T1) spinal vertebral foramens were exposed, and various segments of the BP were identified and harvested (as described previously in Singh et al). 18 Bilateral tibial nerves from these animals were also harvested using a lateral approach. The freshly harvested tissue was preserved in 1% bovine serum albumin (BSA) until testing which was performed within 2 hours from harvesting.

### Mechanical Test Setup

**Fig. 1:**
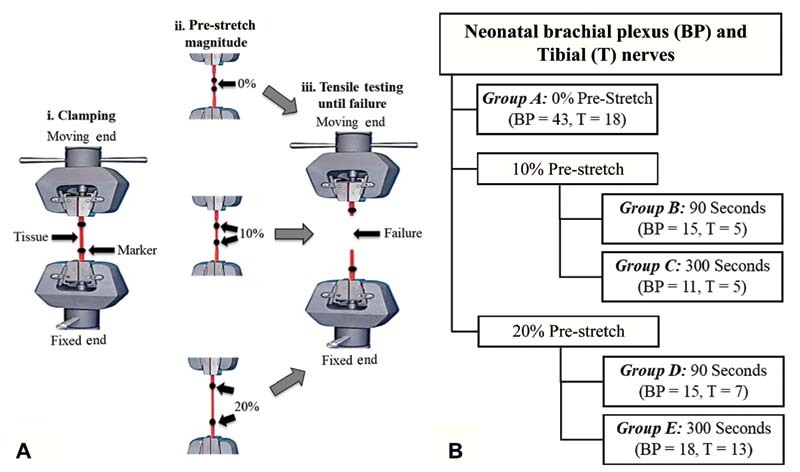
( **A** ) Schematic of the experimental setup including the uniaxial tensile testing machine and ( **B** ) diagram detailing the sample size for the various experimental groups.

An ADMET material testing machine (eXpert 7600, ADMET Inc., Norwood, Massachusetts, United States) was used to perform stress-relaxation and biomechanical tensile testing of the BP and tibial nerves. As shown in Fig. 1A , the harvested nerve was anchored to the testing setup between the fixed and moving ends using specially designed and fabricated clamps. 22 The moving end (actuator end) had a 200 N capacity load cell (500 series, ADMET Inc.) that measured the load subjected on the nerve during stretch.

### Camera System Setup for Strain Measurement

Two fiducial markers using Indian-ink markers (shown by two dots on the tissue in Fig. 1A ) were placed on each nerve near the clamps just prior to testing and a Basler acA640–120uc high-speed video camera (Basler Inc., Exton, Pennsylvania, United States), which collected data at 120 fps (resolution: 658 × 492 pixels), was positioned in front of the material testing machine to capture the images of the nerve during the stretch.

### Testing Procedures

BP and tibial nerve samples were divided into five groups: group A: 0% prestretch, group B: 10% prestretch for 90 seconds, group C: 10% prestretch for 300 seconds, group D: 20% prestretch for 90 seconds, and group E: 20% prestretch for 300 seconds ( Fig. 1B ). A minimum of five BP and tibial nerve samples were tested in each of the five groups. A digital microscope was used to obtain images of the harvested BP and tibial nerves before clamping (at 5X; Digital VHX Microscope, Elmwood Park, New Jersey, United States). A 2-mm ruler (Leitz, Ernst-Leitz-Wetzlar GmbH, Germany) was used during imaging at the same magnification (at 5X) to measure the tissue diameter.

The two clamps were initially set at a distance of 10 to 20 mm (depending on the initial length of the tissue) and the test sample was then clamped with no initial tension prior to stretch, using the built-in GaugeSafe software (ADMET Inc.). The test samples were prestretched to 10 or 20% strain, calculated from the original length of the tissue between the two clamps. The tissue was then held at the assigned prestretch strain level for 90 seconds or 300 seconds. Nerves in group A were not subjected to prestretch and hence, directly underwent failure testing at a displacement rate of 10 mm/second. Nerves in groups B to E were subjected to their respective prestretch values (10 or 20%) and durations (90 or 300 seconds). The prestretched tissue was then subjected to failure testing at a displacement rate of 10 mm/second. During failure, testing, time, load, and displacement data were acquired at a sampling rate of 1,000 Hz. After completion of the experiment, the clamps were checked for presence of tissue, and no tissue in the clamps implied that the tissue had completely slipped, and the results of those experiments were discarded.

### Data Analysis

Load readings were converted to nominal stresses (i.e., load/original cross-sectional area of the sample). Actuator displacement and video data were used to calculate the tensile strain exerted during testing:

ε(t) = (L _f_ − L _i_ )/ *L _i_* , (1)

where *L _i_* is the initial length of the sample while *L _f_* is the final length. The load–displacement and stress–strain curves were plotted, and the failure stress and corresponding strain (i.e., failure strain) were determined. All data analyses were performed using custom codes 23 24 created in MATLAB R2018b (The MathWorks Inc., Natick, Massachusetts, United States) and GraphPad Prism (v9.0, GraphPad Software Inc., La Jolla, California, United States).

### Statistical Analysis

Statistical analysis was performed using SPSS Statistics software (V. 26, IBM Corporation, Armonk, New York, United States). Comparisons between prestretch durations (90 and 300 seconds) and tissue type (BP and tibial) were conducted using independent *t* -tests and between prestretch strain levels (0% [failure only], 10, and 20%) using one-way analysis of variance (ANOVA; post hoc: Tukey's (honestly significant difference) test with correction for unequal sample sizes) if the equal variances assumption was met or Welch's ANOVA (post hoc: Games–Howell) if unmet. A *p* -value of less than 0.05 was considered significant. All values were expressed as mean ± standard error of mean (mean ± SEM).

## Results

**Fig. 2:**
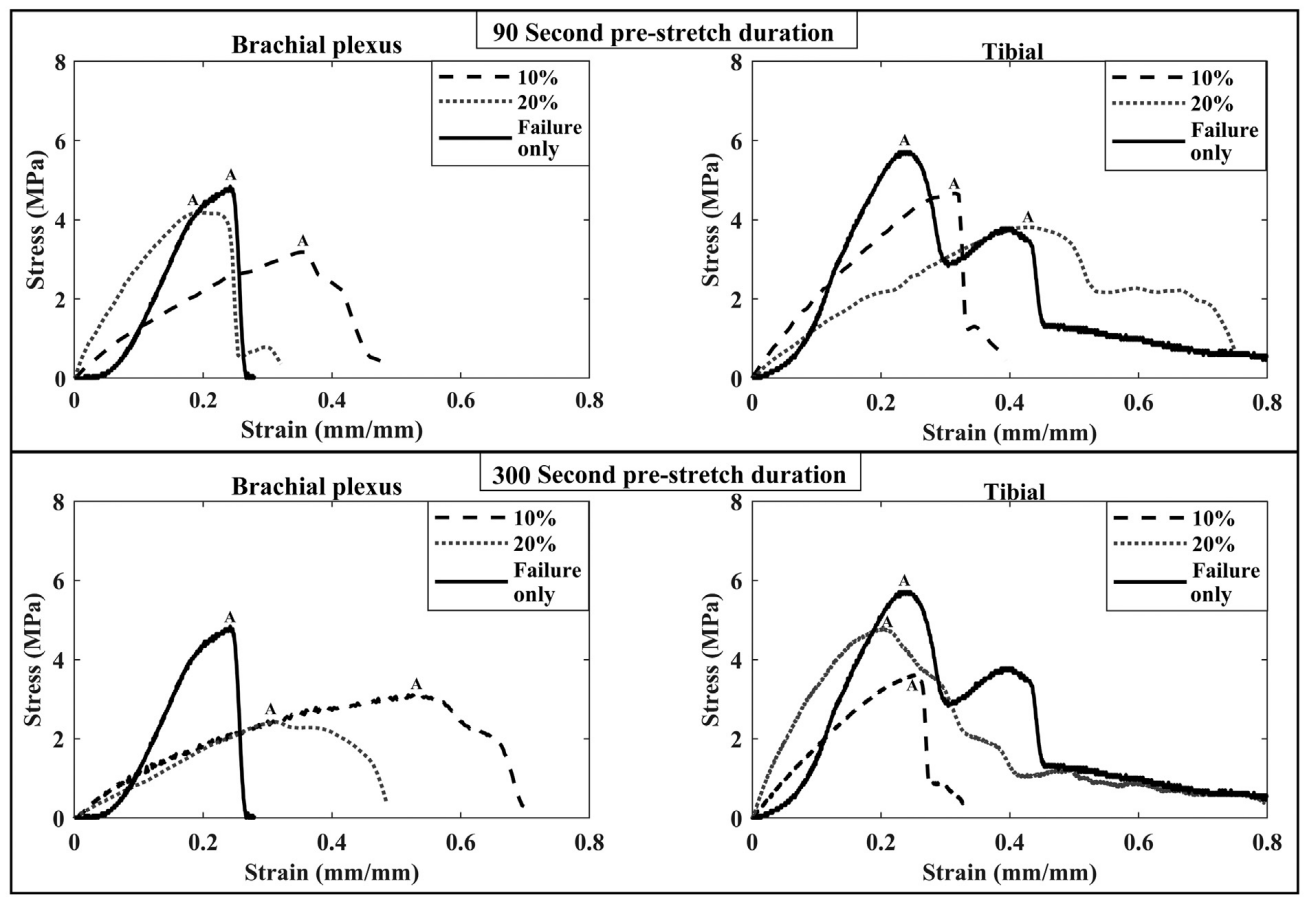
Exemplar stress–strain responses obtained during failure tensile testing of neonatal brachial plexus (BP) and tibial nerves that were subjected to no prestretch (failure only) or prestretch to 10% or 20% strain for 90 (top) or 300 seconds (bottom). Label A for each curve indicates failure stress and the corresponding failure strain.

In group A, a total of 41 BP and 17 tibial nerves did not slip during failure tensile testing. In groups B to E, 58 BP and 28 tibial nerves did not slip during stress relaxation testing. These prestretched samples were then subjected to failure tensile testing. Details of the number of samples that underwent failure testing without slippage in the no prestretch (group A) and post-prestretch (groups B–E) groups are summarized in Table 1 . Figure 2 represents exemplar stress–strain responses observed for BP and tibial nerves in various experimental groups. A typical stress–strain response in all the tested tissue included an ascending slope representing the elastic region, followed by a peak referenced as maximum stress where the nerve rupture was commonly observed (point A in Fig. 2 ), and then a descending slope where stepwise rupture was observed.

**Table 1:** Details of the number of brachial plexus (BP) and tibial nerve samples per experimental groups that underwent failure testing without slippage

Nerve tissue	Group A: failure only	Group B: 10% (90 seconds)	Group C: 10% (300 seconds)	Group D: 20% (90 seconds)	Group E: 20% (300 seconds)
BP	41	15	11	15	17
Tibial	17	5	6	5	12

Note: For groups B to E, the magnitude and duration of prestretch are also shown. For example, 10% refers to the prestretch strain level and 90 seconds refers to the duration of prestretch.

### Brachial Plexus Failure Responses: Effects of Prestretch Magnitude

**Fig. 3:**
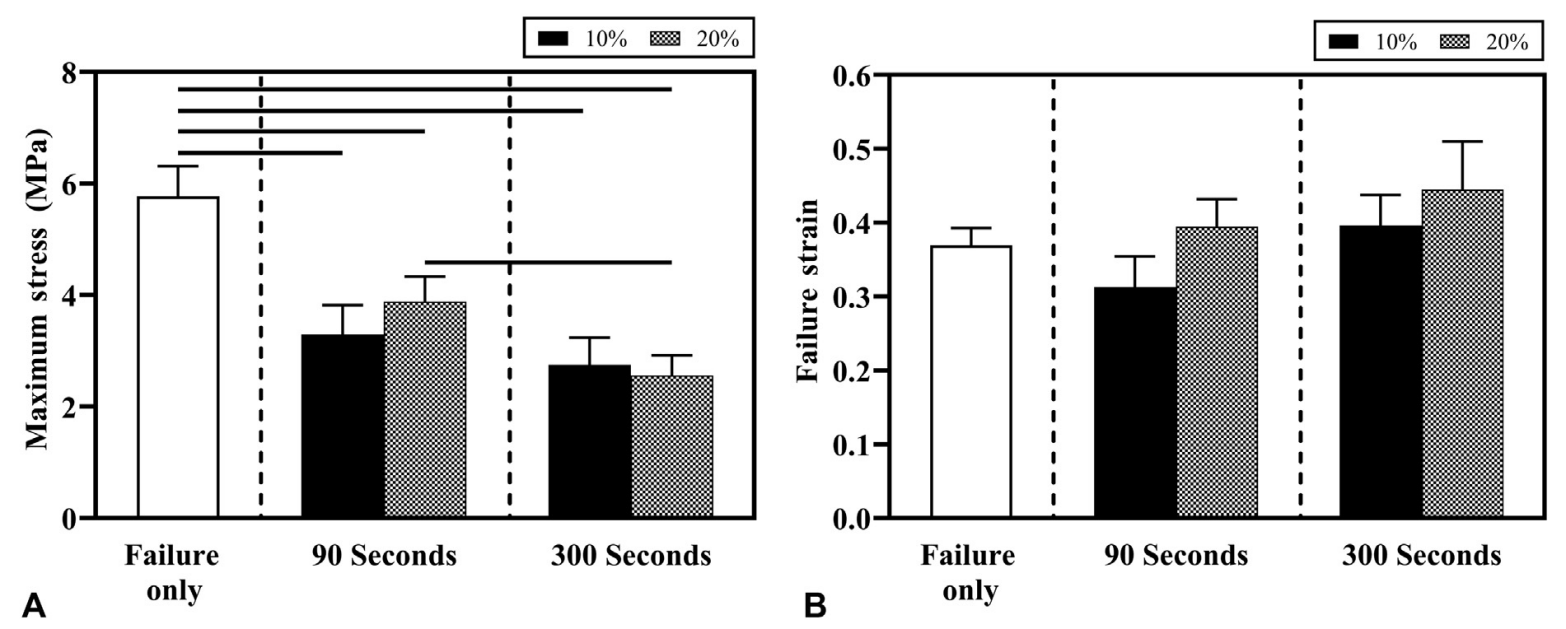
Mean ± standard error of mean (SEM) values of ( **A** ) failure stress and ( **B** ) failure strain observed for brachial plexus (BP) nerve in various experimental groups when subjected to failure tensile testing. SEM values are shown as error bars. Significant differences ( *p <* 0.05) are indicated using a horizontal solid line above the bars.

The failure stress for BP nerves in failure only 0% prestretch (group A) was 5.8 ± 0.5 MPa (mean ± SEM). The failure stress for BP nerves prestretched to 10% strain for 90 seconds (group B) was 3.3 ± 0.5 MPa, and 2.7 ± 0.5 MPa for 300 seconds (group C). The failure stress observed for BP nerves prestretched to 20% strain for 90 seconds (group D) was 3.8 ± 0.4 MPa, and 2.6 ± 0.4 MPa for 300 seconds (group E). Significant differences ( *p* < 0.05) in the failure stress values were observed between the failure-only group (group A, 0% prestretch) when compared with the 10% (groups B and C) and 20% (groups D and E) prestretch groups at both prestretch durations ( Fig. 3A ). However, there was no statistical difference in failure strain between these groups ( Fig. 3B ).

### Brachial Plexus Failure Responses: Effects of Prestretch Duration

No significant differences in the failure stress values were observed between the 90 (group B) and 300 seconds (group C) prestretch groups at 10% prestretch. Significant differences were observed in the failure stress values between BP nerves prestretched to 20% strain for 90 seconds (group D) and those prestretched to 20% strain for 300 seconds (group E; Fig. 3A ). Also, there were no significant differences in failure strain values between the 90 (groups B and D) and 300 seconds (groups C and E) prestretch groups at either prestretch strain level ( Fig. 3B ).

### Tibial Failure Responses: Effects of Prestretch Magnitude

**Fig. 4:**
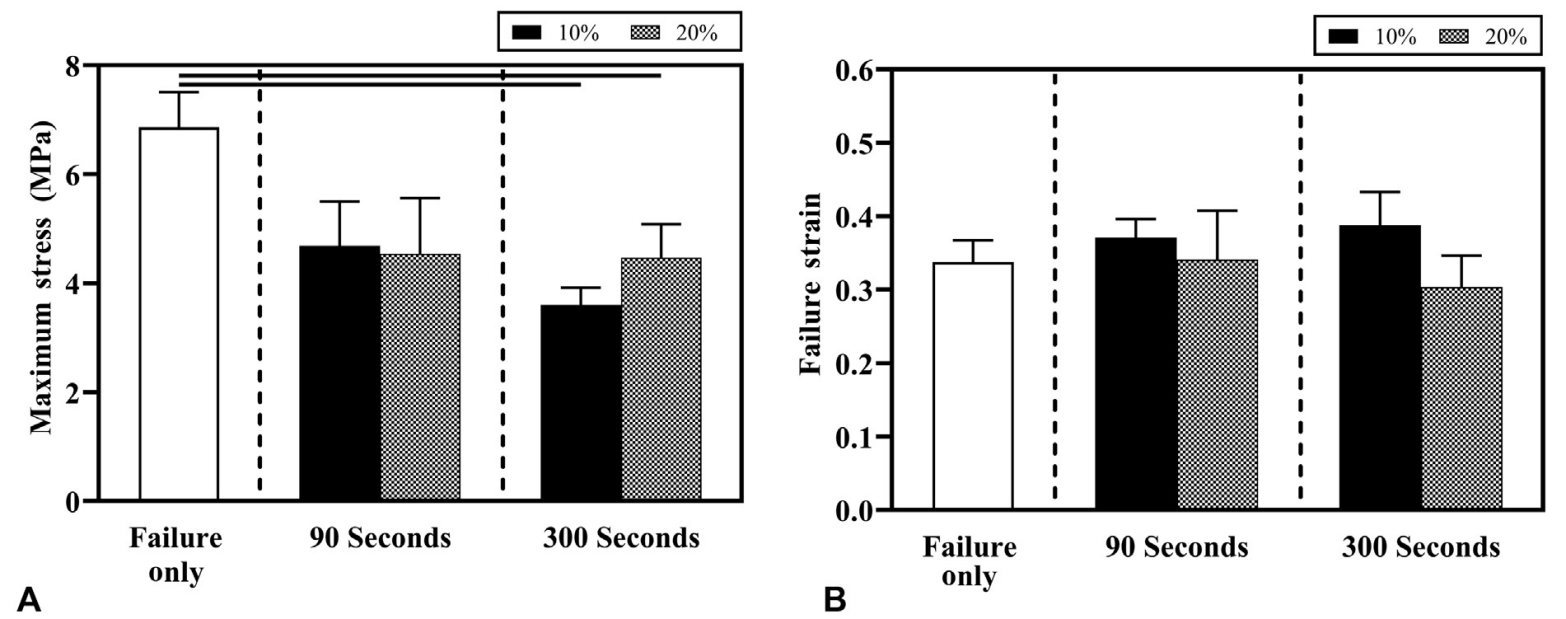
Mean ± standard error of mean (SEM) values of ( **A** ) failure stress and ( **B** ) failure strain observed for tibial nerve in various experimental groups when subjected to failure tensile testing. SEM values are shown as error bars. Significant differences ( *p <* 0.05) are indicated using a horizontal solid line above the bars.

The failure stress for tibial nerves in failure only 0% prestretch (group A) was 6.9 ± 0.6 MPa. The failure stress for tibial nerves prestretched to 10% strain for 90 seconds (group B) was 4.6 ± 0.8 MPa, and 3.6 ± 0.3 MPa for 300 seconds (group C). The failure stress observed for tibial nerves prestretched to 20% strain for 90 seconds (group D) was 4.5 ± 1.0 MPa, and 4.5 ± 0.6 MPa for 300 seconds (group E). Significant differences in the failure stress values were observed between the failure-only group (group A, 0% prestretch) when compared with the 10% (group B) and 20% (group D) prestretch groups at the 300 second prestretch duration, but not 90 seconds ( Fig. 4A ). Also, there was no statistical difference in failure strain between these groups ( Fig. 4B ).

### Tibial Failure Responses: Effects of Prestretch Duration

No significant differences were observed in the failure stress ( Fig. 4A ) and strain ( Fig. 4B ) values between the 90 (groups B and D) and 300 seconds (groups C and E) prestretch groups at either prestretch strain level.

### Comparing Failure Responses of Prestretched Brachial Plexus and Tibial Nerves

**Fig. 5:**
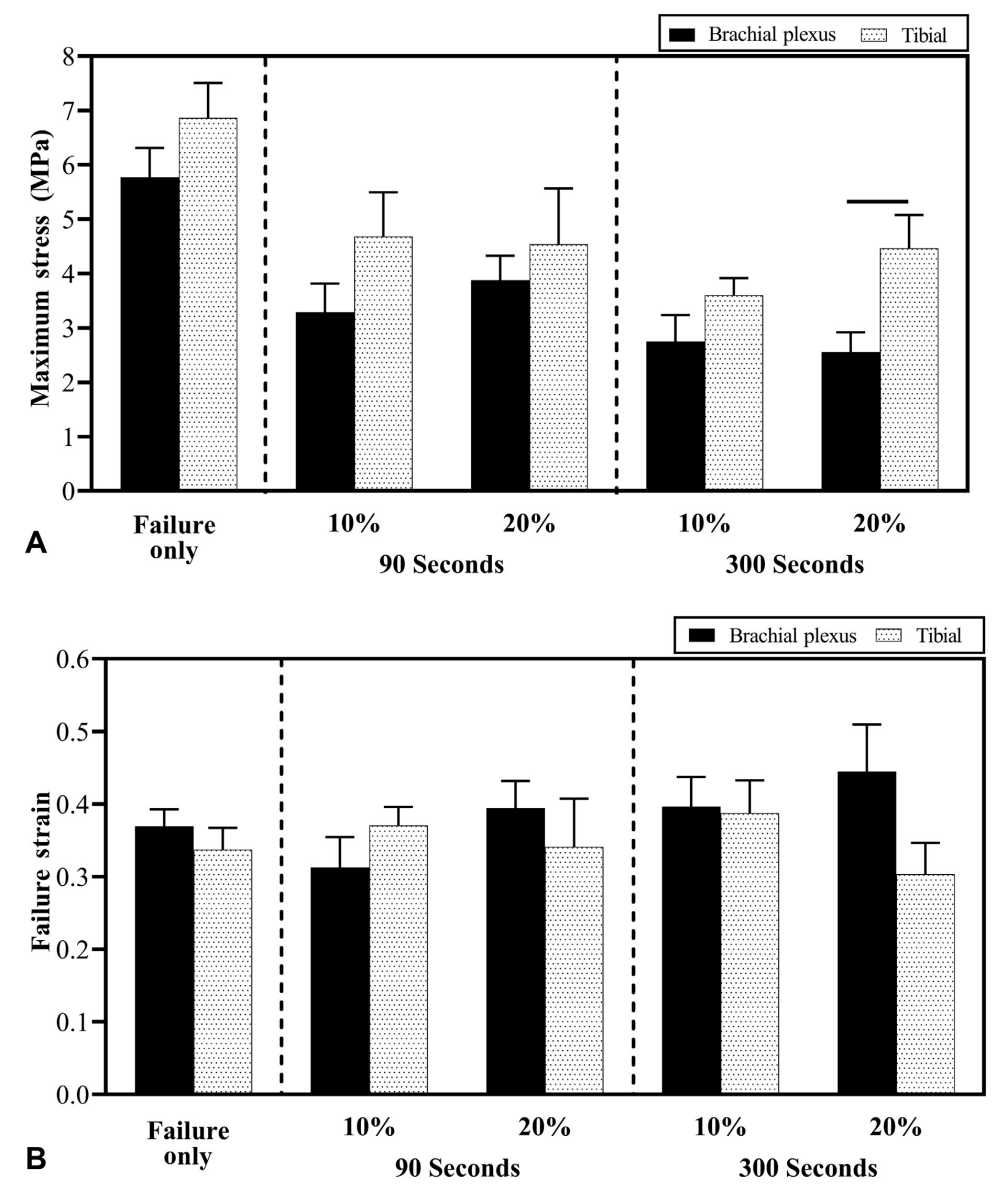
Comparison of biomechanical failure responses of neonatal brachial plexus (BP) and tibial nerve for the various experimental groups. Mean ± standard error of mean (SEM) values of ( **A** ) failure stress and ( **B** ) failure strain observed during failure tensile testing. SEM values are shown as error bars. Significant differences ( *p <* 0.05) between nerve tissues are indicated using a horizontal solid line above the bars.

Significantly higher failure stress was observed in the neonatal tibial nerve (4.5 ± 0.6 MPa) than in BP (2.6 ± 0.4 MPa) when subjected to 20% prestretch for 300 seconds ( Fig. 5A ). No other significant differences in failure stress and strain values were observed between BP and tibial nerve in the other experimental groups.

## Discussion

Stretch-related peripheral nerve injuries cause considerable disability and impose life-long social and economic burdens on the affected individuals. 25 26 To develop preventative strategies, a deeper understanding of factors that modulate nerve injury thresholds is warranted. Since existing studies using adult and neonatal peripheral nerve tissue have primarily focused on biomechanical failure responses when subjected to varying loading rates, the effects of prestretch magnitude and duration on nerve failure responses remain poorly understood. 8 In the current study, we measured the failure stress and strain in neonatal piglet BP and tibial nerve immediately after stress relaxation testing at two different prestretch strain levels (10 and 20%) and durations (90 and 300 seconds). The goal of this study was to report the biomechanical failure responses of prestretched neonatal peripheral nerves to better understand nerve injury mechanisms.

When compared with previously reported failure stresses (1.3–3.5 MPa) based on tensile testing (0.167 mm/second) of adult human cadaveric BP tissue, the failure stress data from the current study on neonatal piglet BP (group A: 5.8 ± 0.5 MPa) are considerably higher. 14 This difference is likely due to the higher loading rate (10 mm/second) used in this study. Another study by Rydevik et al, 1 at similar loading rates, reported failure stress and strain of 11.7 ± 0.7 MPa and 39 ± 2%, respectively, in an adult rabbit tibial nerve. The failure stress (6.9 ± 0.6 MPa) data reported in this study for the neonatal tibial nerve are considerably lower than those reported in the adult rabbit tibial nerve. Such differences in peripheral nerve failure responses between adult and neonatal animal studies warrant careful considerations in translating biomechanical data obtained from adult tissue to neonates.

In the current study, we observed a significant effect of prestretch magnitude on failure stresses for both prestretch durations and nerve types. The reported findings indicate that BP nerves prestretched to >10% strain for 90 or 300 seconds fail at a significantly lower stress when compared with those directly subjected to tensile failure (at 0% prestretch). Similar results were obtained for tibial nerve, but significant differences were limited to the 300-second prestretch duration. Together, these results suggest that neonatal peripheral nerves prestretched to strains >10% for durations of >90 seconds exhibit lower biomechanical injury thresholds and therefore are more susceptible to injury. Future studies should investigate histological changes in prestretched peripheral nerves and help identify structural non-homogeneity that may contribute to the lower mechanical failure threshold after prestretch to strains of varying magnitude.

Previous studies have only reported the long-term (>30 minutes) stress relaxation responses of prestretched peripheral nerves. 27 28 29 30 31 32 However, to account for delivery times that are commonly associated with complicated birthing scenarios, such as shoulder dystocia, investigating the effects of short-term (<300 seconds) prestretch on peripheral nerve biomechanical responses is warranted. 33 34 35 36 37 In this study, two different prestretch durations, 90 and 300 seconds, were chosen. The reported findings indicate that BP nerves prestretched to 20% strain for 300 seconds failed at a significantly lower stress than those prestretched for 90 seconds. These results suggest that nerve prestretched to higher strains for longer durations predisposes neonatal BP tissue to mechanical injury. The clinical relevance of this finding is significant, especially in cases of shoulder dystocia wherein a prolonged head-to-body delivery time has been associated with increased risk of complications, such as BP injury. 33

In this study, we also compared the failure response of neonatal BP nerve to that of tibial nerve to explore the regional variability in peripheral nerve failure properties, especially when prestretched. The finding that neonatal BP nerve failed at a significantly lower stress than tibial nerve when subjected to a prestretch of 20% for 300 seconds is of importance. A similar trend, although not statistically significant, is also notable in other testing groups, and likely reflects differences in underlying nerve fiber arrangement as previously reported in adult animal studies. 1 Peripheral nerves have a wavy, undulating structure that allows for some degree of physiological stretch. 1 38 39 Using human cadaveric tissue, Kerns et al 39 reported differences in undulations patterns at failure between tibial and peroneal nerves. The significantly lower failure stress seen in BP nerve when compared with tibial nerve, in this study, suggests that the BP nerve may have fiber orientations that present a biomechanical disadvantage. Future histological studies are warranted to confirm any structural differences that might play a role in the tensile loading and mechanical responses of the nerves.

## Limitations

The lack of histological processing of the tested samples represents a major limitation of this study. Visualizing structural changes in prestretched nerve tissue may be predictive of gross tissue mechanical response. Future work investigating histological changes that occur in prestretched nerves is therefore necessary to fully understand the impact of nerve prestretch on injury patterns. 40 Although all efforts were made to standardize testing environments, 41 uncontrolled differences in subtle aspects of testing, such as tissue dissection, and inherent variation in animals may have also influenced the biomechanical results.

## Conclusion

In conclusion, the current study is the first to report the failure responses of neonatal BP and tibial nerves when subjected to varying magnitudes and durations of prestretch. The reported data provide novel insights into nerve injury thresholds that can be incorporated in existing teaching and future 40 41 42 43 44 45 46 47 48 finite element and computational models of neonatal peripheral nerve injury. Future studies investigating the effects of prestretch magnitude and duration on functional outcomes using an in vivo model will provide further insight 49 into stretch-related peripheral nerve injury mechanisms and extend the translational scope of the current findings.
